# The Role of Rituximab in Primary Central Nervous System Lymphoma

**DOI:** 10.1007/s11912-020-00941-8

**Published:** 2020-06-29

**Authors:** Jacoline E. C. Bromberg, Matthijs van der Meulen, Jeanette K. Doorduijn

**Affiliations:** 1 Departments of Neuro-Oncology, Erasmus MC Cancer Center, Postbus 2040, 3000 CA Rotterdam, The Netherlands; 2 Departments of Hematology, Erasmus MC Cancer Center, Postbus 2040, 3000 CA Rotterdam, The Netherlands

**Keywords:** PCNSL, Rituximab, Treatment, Central nervous system, Lymphoma

## Abstract

**Purpose of Review:**

The treatment of primary central nervous system lymphoma (PCNSL) is still under debate. One of the issues is the role of rituximab in improving the outcome. Here, we summarize the existing evidence, and comment on the literature on this topic.

**Recent Findings:**

Two randomized controlled studies have been published recently, with conflicting results. Although the evidence of the benefit of rituximab is limited, it is already incorporated into many treatment regimens, both in studies and in standard clinical practice.

**Summary:**

The use of rituximab in PCNSL is still a matter of debate. A positive effect on the outcome is uncertain. However, there are no clinical signs of significantly increased toxicity. The uncertain positive effect should therefore be weighed against the increased costs of the treatment.

## Introduction

Primary central nervous system lymphoma (PCNSL) is a rare aggressive lymphoma localized in the brain, leptomeninges, or eye, with no extra CNS manifestations. The large majority, more than 90% of cases, have the morphology and phenotype of a diffuse large B cell lymphoma (DLBCL) [1]. PCNSL accounts for < 1% of all non-Hodgkin lymphomas and 3% of all brain malignancies, but the incidence is reported to be rising especially among older patients; the age-standardized incidence is 0.4–0.5 per 100,000 per year [2, 3]. Because of its rarity, very little high-quality evidence regarding treatment is available. Only 2 randomized phase III studies and 5 randomized phase II studies have been performed, one of which included only 52 patients [4, 5••, 6••, 7–10]. The treatment regimen most commonly used in systemic DLBCL, R-CHOP chemotherapy, has been shown to be ineffective in PCNSL, likely because of its inability to penetrate the blood-brain barrier (BBB) [11]. High-dose intravenous methotrexate (HD-MTX) can penetrate the BBB and is considered the cornerstone of the treatment of PCNSL. Although combination regimens are generally used, there is no consensus regarding the other agents used in combination with HD-MTX, and several regimens have been published, e.g., combination with procarbazine and vincristine (MPV), with temozolomide, with carmustine and teniposide/etoposide (MBVP), or with high-dose cytarabine [7, 12–14]. The only randomized trial investigating the benefit of combination therapy in comparison with HD-MTX alone was the IELSG 20 study, comparing HD-MTX monotherapy every 3 weeks with the combination of HD-MTX/HD cytarabine, which showed a better outcome after the combination therapy [7]. The response rate of all these regimens is generally high, in many studies 80–90% with complete response rates varying between 55 and 75%. Unfortunately, despite consolidation treatment with whole-brain radiotherapy or autologous transplantation, many patients relapse.

## Rituximab

The relatively poor outcome of PCNSL with chemotherapy compared with systemic DLBCL asked for a better induction regimen. In systemic DLBCL, a significant improvement has been achieved with the introduction of rituximab. Rituximab is a chimeric monoclonal antibody that targets the CD20 cell surface protein. In combination with CHOP, it has improved the event-free (EFS) and overall survival (OS) by 15–20% and is part of standard of care. [15, 16]

The role of rituximab in PCNSL, however, is less clear. Due to its large size (145 kD), it is questionable whether it can pass the BBB, and CSF concentration after intravenous administration has been shown to reach only 0.1% of the serum concentration. [17] However, at the site of the PCNSL infiltration, the customary homogeneous enhancement with gadolinium contrast agent suggests that the BBB is generally disrupted, at least at presentation. In patients with active leptomeningeal disease, the CSF concentration of rituximab was 3–4% of the serum concentration, suggesting that disruption of the BBB allows at least some penetration [18]. In recent years, many studies have been published regarding the efficacy of rituximab in PCNSL including 2 randomized studies, though with conflicting results. Here we summarize and discuss the available evidence in order to aid clinical decision-making.

Several retrospective and single-arm prospective studies have been published of which many [19–29], but not all [24, 30–32], suggested improved progression-free survival with rituximab in comparison with historic controls or previously published results without rituximab (Table 1). All these studies reported that combination treatment is feasible. For example, an early phase II study, combining HD-MTX, procarbazine, and vincristine with rituximab, followed by low-dose whole-brain radiotherapy (WBRT) resulted in the intention-to-treat population in a median PFS of 3.3 years and 2-year progression-free survival (PFS) of 57% [23]. A retrospective study in 120 PCNSL patients, of which 18 patients received additional rituximab, reported age > 60 years, ECOG PS > 1, and elevated LDH as risk factors for poor survival, whereas cytarabine and rituximab in the treatment regimen were factors predicting improved survival, though only in univariate analysis [24]. On the other hand, a large multicenter retrospective registry study found improved response rate but no survival benefit after adding rituximab to MPV and cytarabine [31]

**Table 1:** Published reports regarding rituximab in primary CNS lymphoma

Author, year	No. of pts (N with/wo R)	Study type	Regimen	PFS (months)	OS (months)	Comparator	Conclusion
Chamberlain, 2010	40 (40/0)	PPII-MC	R-HD-MTX	Med 21	Med 29	NOA3 study	Improved results compared with prior studies with HD-MTX only
Fritsch, 2011	28 (28/0)	PPII-SC	R-MCP	Med 16	Med 17.5	MCP	Improved PFS not OS
Birnbaum, 2012	36 (36/0)	RSC	R-MTX-IFO	6 months 94%	6 months 63%	MTX-IFO	Improved response rate and PFS
Morris, 2013	52 (52/0)	PPII-SC	R-MPV-A	2 years 57%	2 years 81%	MPV-A	Improved response rate and survival
Gregory, 2013	120 (18/99)	RMC	MTX-based			MTX-based	R favorable in univariate not multivariate analysis
Holdhoff, 2014	81 (27/54)	RSC	R-HDM-TX	Med 4.5	Med 26	HDM-TX	Improved response rate and PFS
Kansara, 2015	74 (25/49)	RMC	R-MTX	5 years 38%	5 years 17%	MTX	No difference
Madle, 2015	81 (81/0)	RSC	Various	3 years 78%	3 years 40%	Various	R predictive of better OS
Mocikova, 2016	164 (49/115)	RMC	R-MPV-	Med 23 vs 11	Med 29 vs 19	MPV	R predictive of PFS not OS
Ferreri, 2016	219*	RCT-PII-MC	R-MTX-AraC	2 years 46%	30 months 52%		Improved response rate and PFS especially in MATRIx group
Sun, 2017	60 (24/36)	RMC	RMAD	Better with R	No difference	MAD	Longer PFS not OS
Houillier, 2017	90 (90/0)	RMC	R-MPV-C	Med 12 vs 9.7	Med 37 vs 17	MPV-C	Higher response rate (77% vs 53%) not survival
DaBroi, 2018	57 (18/39)	RMC	R-MPV	Med 34/12	Med 46/15	MPV/nordic	Multivariate analysis no difference
Swinnen, 2018	26 (26/0)	PPII	R-MPV?	Med 34	Med > 40	RTOG 93–10	Low number of patients
Bromberg, 2019	199 (99/100)	RCT-PIII-MC	R-MBVP-C	2 years 54%	2 years 71%		No difference
Chen, 2019	62 (62/0)	RSC	R-MT	2 years 81% vs 46%	2 years 82 vs 66%	MT	R-MT better

*PPII-MC*, prospective phase II multicenter; *PPII-SC*, prospective phase II single center; *RSC*, retrospective single center; *RMC*, retrospective multicenter; *RCT-PII-MC*, randomized controlled trial phase II multicenter; *RCT-PIII-MC*, randomized controlled trial phase III multicenter

*69 pts. with rituximab, 75 with rituximab and thiotepa, and 75 without rituximab

Two prospective, randomized studies, the IELSG 32 and the HOVON 105/ALLG NHL 24, have been published investigating the effect of rituximab in PCNSL.

The IELSG 32 was a double-randomized, phase II study investigating both induction treatment and consolidation treatment. In this study, 227 patients aged 18–70 years (median 57 years) were first randomized 1:1:1 for HD-MTX and cytarabine (arm A); HD-MTX and cytarabine, and rituximab (arm B), or HD-MTX; and cytarabine and rituximab and thiotepa (arm C, MATRix regimen) [6••]. Patients responding or with stable disease were subsequently randomized for consolidation with either WBRT or autologous stem cell transplantation (ASCT). Two hundred nineteen patients were assessable for the first randomization. The primary endpoint for the first randomization was complete response (CR) rate; the secondary endpoint was PFS. Only arm C (MATRix regimen) achieved the minimum CR rate (> 45%) considered of interest by the authors. Arm A showed a 23% CR rate, and arm B had a CR rate of 30%. Patients in arm C (MATRix) also had the best 2-year PFS, 61% vs 46% (arm B), and 36% (arm A). The 2-year overall survival rate was 42%, in group A, 56% in group B, and 69% in group C. The conclusion of the authors is that the MATRix regimen should be the standard chemoimmunotherapy treatment for patients up to 70 years old with a PCNSL. However, the question remains what the contribution of rituximab is in this regimen since no arm investigated the efficacy of thiotepa without rituximab. Treatment results in arm A, which is identical to the experimental arm in the previous study utilizing this regimen, are considerably worse than expected from the previous study (23% compared with 46% CR rate) [7]. With a poorly performing comparator arm, the true value of the addition of rituximab (arm B) is difficult to discern. A second limitation of the study is the differing consolidation treatments given after the second randomization. Differing effects of the two consolidation therapies, although only given after response assessment and thus not affecting the primary endpoint (CRrate), may also have influenced survival results despite their random allocation.

The HOVON 105/ALLG NHL 24 study was a randomized phase III study investigating the effect of rituximab in PCNSL [5••]. In this study, 200 patients with newly diagnosed PCNSL aged 18–70 (median 61) years old were randomized 1:1 for treatment with MBVP chemotherapy with or without rituximab. Responding patients were subsequently treated with HD cytarabine, and patients under 61 years old were in addition consolidated with low-dose (30 Gy) WBRT with an integrated boost to the tumor area in case of less than CR/CRu. The primary endpoint in this study was a 1-year event-free survival (EFS), where EFS was defined as either not attaining CR/CRu, progression, relapse, or death. With this endpoint, treatment given to patients off protocol, which may vary according to center, e.g., elderly patients treated with radiotherapy after not attaining CR/CRu, will not influence the result.

EFS at 1 year was 49% without and 52% with rituximab, with a hazard ratio (HR) of 1.0 (95% CI 0.7–1.43), *p* = 0.99, thus showing no effect of rituximab on EFS. Similarly, 1-year PFS did not differ between the arms with 58% in the MBVP group and 65% in the R-MBVP group (HR 0.77, 95% CI 0.52–1.13, *p* = 0.18). Thus, contrary to the results in the IELSG 32, this straightforwardly designed study suggests no effect of rituximab. Because of the discrepancy in consolidation treatment between older and younger patients, the authors performed an unplanned subgroup analysis to evaluate a possible difference in the effect of rituximab in these patient groups. Unexpectedly, rituximab appeared to improve event-free survival in younger patients: despite only 47 patients in each arm, the HR for EFS was 0.56 in younger patients with *p* = 0.054. Whether this is a true effect of rituximab in younger patients, possibly related to the radiotherapy which was given in this group, or whether it is just a coincidental finding caused by small patient numbers is uncertain. Such an age effect of rituximab has not been described before and should be investigated further before assuming it is a true effect.

The explanation for the discrepant results between the two randomized studies still remains elusive. In both studies, rituximab was first administered before commencing chemotherapy and/or most intensively in the first cycle to make maximal use of the disrupted blood-brain barrier. In the IELSG study, this was on day − 5 and 0 of each cycle, and in the HOVON 105, it was on days 0, 7, 14, and 21 of the first cycle and 0 and 14 of the second cycle. Interaction between the type of chemotherapy and rituximab may also have influenced results since the combination of agents differed between the two studies, even though, again, such a difference has not been described before. Results in IELSG 32 suggest at least that additional agents may improve effect: the best arm in this study not only incorporated rituximab but also thiotepa. Finally, despite the primary endpoint having been reached in both studies, overall survival results are still immature and longer follow-up may in the future shed a different light on the effect of rituximab on PCNSL.

In order to improve the validity of the data, a systematic review and meta-analysis was performed of randomized studies comparing regimens with or without rituximab in PCNSL [33•]. An extensive literature review showed only the above two prospective randomized studies that enrolled a total of 343 patients (from the IELSG 32 study, only arm A and arm B were included in the analysis). The main endpoints of interest were OS and PFS. For overall survival, the risk of bias was found to be low. The hazard rate (HR) of death in the pooled analysis was 0.76 (95% CI, 0.52–1.12), thus showing no statistically significant evidence for an OS benefit of the addition of rituximab. In the meta-analysis, the HR for PFS was 0.65 (95% CI, 0.45–0.95), suggesting a possible benefit of rituximab, but with low certainty, because there is a risk of bias in the assessment of PFS; because of the unusually poor results in the control arm of the IELSG 32; and, the last but not least, because of the unexplained heterogeneity of the studies.

In addition to survival endpoints, it is also important to measure toxicities and the effect of treatment regimens on neurocognitive functioning; health-related quality of life; and radiological changes, such as white matter abnormalities (WMA). Clinical toxicities (e.g., hematological, nephrotoxicity, and hepatotoxicity) were similar in those treated with and without rituximab in both clinical trials [5••, 6••]. Both neurocognitive functioning and health-related quality of life scores were similar in both arms of the HOVON 105/ALLG NHL 24 study [34, 35]. Regarding radiological changes, one single-center cohort (*n* = 47) study showed that those treated with rituximab in combination with HD-MTX developed more WMA (46% vs 68%) than those treated with HD-MTX alone. Moreover, The WMA were detected sooner in those treated with rituximab (2.8 vs 10.7 months) [36]. However, these results are unexpected and need confirmation in other and larger populations.

## Current Practice

The IELSG 32, first randomization, was published in 2016. The HOVON 105/ALLG NHL 24 study was published in January 2019. Before the results of these studies were known, many physicians and clinical investigators already started to incorporate rituximab in their treatments, based on retrospective data, and hoping for a positive effect, while awaiting the outcome of the randomized studies.

The French Intergroup ANOCEF-GOELAMS initiated a study in 2008 to investigate the consolidation with either whole-brain radiotherapy or autologous stem cell transplantation. The induction regimen was comparable with the HOVON 105/ALLG NHL 24 regimen, MBVP, but including rituximab [9].

The MATRix regimen from the IELSG 32 study is used as the induction regimen in the ongoing study from IELSG and German Cooperative PCNSL study group, comparing consolidation with autologous stem cell transplantation or conventional immune-chemotherapy [37]. This study was registered in 2014.

In the PRIMAIN study in elderly patients, rituximab is combined with methotrexate, procarbazine, and lomustine [38].

Large US groups also have started to use rituximab in and outside (phase II) trials. Examples are the CALGB 50202 (Alliance 50202) recruiting patients from 2004 to 2009 [39] and the R-MPV regimen in the Memorial Sloan Kettering Cancer Center [40].

## Conclusion

Altogether, a positive effect of rituximab in PCNSL has not been proven. In this rare disease, two prospective randomized studies investigating the role of rituximab have been performed, unfortunately with conflicting results. The meta-analysis which was performed could not solve the dilemma. An effect specifically in younger patients, as suggested by the subgroup analysis of the HOVON 105/ALLG NHL 24 study, still awaits confirmation but could perhaps explain part of the discrepancy since patients in the IELSG 32 study were somewhat younger (median 57 years vs 61 years in the HOVON study). Hopefully, when the results of the studies have matured enough to allow reliable evaluation of overall survival, a definitive conclusion can be reached. In the meantime, many physicians and clinical investigators have incorporated rituximab in their treatment regimens. Even with low evidence of a beneficial effect, the relatively low toxicity of rituximab and its customary application in systemic DLBCL has resulted in widespread use. However, rituximab use might be a financial burden, and particularly in situations where costs are important, the choice to refrain from adding rituximab can still be well defended.
